# Obstetrical Cases in Recent Practice

**Published:** 1846-08

**Authors:** A. Alpuente

**Affiliations:** New Orleans


*Obstetrical Cases in recent Practice.* By A. Alpuente, M. D., of New Orleans.

Case 1. *Difficult Labor—Convulsions—enlargement of the os tincæ by incision—success.* On the 4th of April last, about 7 P. M., I was called to visit a female slave in Philippa street. She was stout, and had enjoyed good health. In the night preceding my visit, she was taken with vomiting, and, towards 5 o'clock, with convulsions; on my arrival I found her in the following state. Her pregnancy had arrived at the last stage; the convulsions with which she suffered, partook of the character of epilepsy; they were due, from all appearances, to congestion of the brain, the result of a first labor. On examination, the neck of the uterus was found entirely obliterated, and the os uteri itself barely pervious. Venesection was now resorted to, an anti-spasmodic potion, and a full bath.

At 10 P. M., I visited her again; she was in the same state: there was no dilatation whatever; the contractions of the uterus were strong in the extreme. Two blisters were now applied to the calves of the legs, and an enema of castor oil and assafœtida, administered. After another examination, I requested the aid of another physician, and named Dr. Daret.

At half past 12 I met Dr. Daret; the same symptoms persisted. We now examined the woman with the speculum; the *os tincæ* was reduced to a size barely sufficient to admit a small probe; the neck of the uterus was entirely obliterated; every trace of it had gone, excepting this small aperture which seemed to indicate its position.

After mature deliberation, we concluded that an operation alone could extricate the woman from her great danger, and that immediate delivery was required to put an end to the puerperal convulsions: we demanded, however, the advice of a third physician, and Dr. Guesnard was named. With the exception of the blisters, the same prescription was repeated.

At half past two o'clock we met Dr. Guesnard; at that moment our patient seemed to be in a more favorable situation; the puerperal convulsions had ceased a few moments before our arrival. The speculum was re-applied, but in consequence of the movements of the patient, it was difficult to detect the aperture which marked the seat of the os tincæ; on careful inspection, Dr. Guesnard found the aperture immediately behind the arch of the ossa pubis, and sufficiently dilated to admit the introduction of the finger, by which he was enabled to ascertain that the presentation was that of the vertex. In consequence of these favorable changes, Dr. Guesnard was of opinion that the labor would terminate naturally; every thing in fact, seemed to announce a favorable result. The two consulting physicians then retired, without fixing any hour for future consultation, deeming it unnecessary.

At 5 o'clock I again saw the patient; the puerperal convulsions were very strong, and the *os uteri* was no more dilated than when we left her. Dr. Daret was called again.

At 8 o'clock I again met Dr. Daret; we sent immediately for Dr. Guesnard: the immediate termination of the delivery was now deemed of absolute necessity, since twelve hours had elapsed, and the convulsions still continued.

At 11 o'clock, Dr. Guesnard not having yet been found, and as the convulsions were much stronger, we called on Dr. Tricou. Before the arrival of Dr. Tricou, the convulsions ceased again, and the woman relapsed into about the same state that she was in when Dr. Guesnard first saw her. Dr. Tricou now thought it advisable to postpone all interference for a few hours longer. The extract of belladonna was, however, applied to the *os uteri*, an anodyne enema administered, and venesection again resorted to.

The convulsions soon returned, and at half past 12 o'clock the operation was decided on. The patient was now placed on her back, at the edge of the bed, in the usual position for such operations, and being properly assisted by the attendants, I now introduced through the highly contracted *os tincæ*, the index finger, conducting upon it a probe pointed bistoury, with which I made three incisions, one of about six lines posteriorly, which was directly under the arch of the ossa pubis, and two lateral ones of about four lines each in length ; I then introduced my hand slowly into the uterus, and applied the forceps, as we had agreed upon before the operation. When the head was passing through the os externum, the perineum was put severely on the stretch, which induced us to make a few slight incisions on the posterior part of the *labia pudendi* in order to prevent rupture. The contractions of the uterus was strong, and the placenta was thrown out immediately after the delivery of the child. Unfortunately the child was born dead; we instantly resorted, nevertheless, to all the means which might have contributed to the restoration of life had it been only asphyxiated.

All the nervous symptoms ceased as soon as the delivery was over, but the debility which now ensued was extreme, the pulse was small, and between eighty and ninety. The loss of blood was not greater than in any ordinary accouchement. At half past three o'clock I retired, leaving our patient in as satisfactory a condition as could be expected. The next morning, at our visit, we found her in the followiug state : the skin was dry ; the abdomen distended and very sensible ; general prostration of strength ; the pulse was small and frequent ; there was very little discharge of blood ; there was also retention of urine, which was relieved by the catheter. Poultices were now applied to the abdomen, sinapisms to the lower extremities, and a tonic mixture was ordered. The blisters were also dressed, a hip-bath provided, and a cathartic enema prescribed. By three o'clock a great improvement had taken place; the pulse had become nearly natural, her strength had improved, and she returned rational answers to all our questions. On the 6th, the abdomen had resumed its natural dimensions, the pulse was about the same, her intelligence had revived, although there was still some tendency to collapse. The catheter was now again resorted to, and a hip-bath, poultices, a laxative enema, emollient injections, cooling drinks, and chicken broth prescribed. On the 7th, the satisfactory condition of the patient continued the whole day, but on the 8th, there was some slight tension and tenderness of the abdomen, but the urine had been freely evacuated; her pulse was also frequent. Eighteen grains of calomel were now prescribed, to be followed by the enema; and the hip-baths, the poultices and the injections were ordered to be repeated. About 12 o'clock this day, two clots of blood were discharged, after which a striking improvement took place. At night, however, the abdomen was again painful, and the pulse frequent and full; the *decubitus* was also unfavourable ; there was suppression of the lochial discharge. Sinapisms to the thighs and feet, and a tonic mixture, were now ordered. On the 10th, the pulse was from seventy to eighty, and rather small, the pain had nearly entirely left the abdomen, there was some heaviness of the head, but the evacuations were natural, the countenance was good, and her intelligence had returned; the cooling drinks, the enemata and injections were again prescribed, together with cold lotions to the head, and a hot foot-bath. After the 11th, the improvement of the patient was progressive; slight purgatives were administered from time to time, and at the present moment, she is in the enjoyment of excellent health.

Case 2.—*Premature Delivery of Triplets.*—On the 19th of September, at 11 A. M., I received a call to visit a poor woman, who had been delivered, during the previous night, of three girls. She had not completed the full time of her pregnancy; labor had come on between the seventh and eighth month; during her confinement she had received no assistance whatever, either from midwife or physician.

I immediately repaired to the corner of Esplanade and Claiborne sts., and there I found a woman about 40 years of age, habituated to hard labour. She is the mother of four children, and had, previously to her last confinement, two miscarriages, shortly after which she became pregnant again. On the 18th, she felt unusually fatigued after her daily occupations; she also felt some pains in the back and about the uterus, but did not think assistance of absolute necessity. In the night, however, the pains increased, and at half past 12 she was delivered of a girl, which presented the feet; after the lapse of half an hour, she was delivered of *another girl*, the vertex of which presented; and about a quarter of an hour afterwards, the placenta and membranes came away, enveloping *a third female infant.* All these children were delivered alive; the last, however, survived but a few minutes, the second died at 9 o'clock the next morning, and the first died on the 20th, at 12 o'clock.

The size of the children was as follows:

Length, from the top of the head to extremities of the toes, extended, 16 inches.

Occipito-frontal diameter of the head, 4 inches, 2 lines.

Occipito-mental diameter of the head, 4 inches, 6 lines.

Bi-parietal diameter of the head, 3 inches.

On my first visit, the woman was rather weak; her pulse was small and frequent, the perspiration was profuse, (she was delivered in a feather bed,) the hæmorrhage was moderate, the uterus still tolerably large, and occupying the left hypochondriac region. On examination, the neck of the uterus was soft, and the *os uteri* small—so diminished in diameter, that I could not succeed in passing my fingers, in order to ascertain within, the cause, if any, of its contractions, which I attributed to the extreme distension that it had previously undergone. I prescribed previously to my departure, a tonic mixture, infus. tiliæ, and chicken broth for nourishment. The next day she was better, and continues to do well.

This is the second case of triplets which I have seen in this city.

About two years ago, I was called to visit a woman who had miscarried at the sixth month. In this case there were two male infants enveloped in the same membrane; they were both born dead. The remaining infant was a female, which was contained in a separate sack; it lived but a few minutes. My services were required on account of the attending hæmorrhage, but the recovery of the patient was speedy.—*N. O. Med. and Surg. Journ.*

